# *Notes from the Field:* Characteristics of E-cigarette, or Vaping, Products Confiscated in Public High Schools in California and North Carolina — March and May 2019

**DOI:** 10.15585/mmwr.mm6942a7

**Published:** 2020-10-23

**Authors:** Mays Shamout, Lauren Tanz, Carolyn Herzig, Lisa P. Oakley, Corey M. Peak, Amy Heinzerling, Marisa Hast, Eileen McGowan, Rebecca J. Williams, Catherine Hess, Chunxia Wang, Sarah Planche, Sally Herndon, Jim Martin, Susan M. Kansagra, Maeh Al-Shawaf, Paul Melstrom, Kristy Marynak, Michael A. Tynan, Israel T. Agaku, Brian A. King

**Affiliations:** ^1^Office on Smoking and Health, National Center for Chronic Disease Prevention and Health Promotion, CDC; ^2^Epidemic Intelligence Service, CDC; ^3^Division of Public Health, North Carolina Department of Health and Human Services; ^4^Center for Healthy Communities, California Department of Public Health; ^5^Division for Parasitic Diseases and Malaria, Center for Global Health, CDC; ^6^Oak Ridge Institute for Science and Education, Oak Ridge, Tennessee; ^7^Institute for Population Health Improvement, University of California Davis Health System, Sacramento, California; ^8^Tobacco-Use Prevention Education Office, California Department of Education, Sacramento, California.

E-cigarette, or vaping, products are electronic devices that produce an inhalable aerosol by heating an e-liquid that typically contains nicotine and other additives (*1*). Nicotine is highly addictive, can harm adolescent brain development, and can prime the brain for addiction to other drugs (*1*). In 2019, 27.5% of U.S. high school students currently used e-cigarettes (*2*), and 73.4% of high school students had observed e-cigarette use on school grounds (*3*). E-cigarette use among U.S. youths increased considerably during 2017–2019 (*2*). This rise coincided with the increased popularity of “pod mods,” which are products with a prefilled or refillable pod cartridge (pod) and a modifiable (mod) system. Pod mods typically use nicotine salts rather than the freebase nicotine used in most other e-cigarette, or vaping, products and conventional tobacco products (e.g., cigarettes).* Nicotine salts, which have a lower pH than freebase nicotine, allow particularly high levels of nicotine to be inhaled more easily and with less irritation to the throat than freebase nicotine.^†^ The most commonly sold pod mod brand is JUUL, which accounted for 75% of all U.S. e-cigarettes sales by the end of 2018.^§^ A majority (59.1%) of U.S. high school student e-cigarette users report JUUL is their usual brand (*2*).

To understand the types of e-cigarette, or vaping, products used on school grounds, CDC conducted an environmental assessment in California and North Carolina public high schools in March and May 2019, respectively. An e-mail request from the California Department of Public Health and Department of Education and North Carolina Division of Public Health was sent to a convenience sample of 1,456 California high schools and a state-representative sample of 25 North Carolina high schools to request available products confiscated from students or found on school grounds during the 2018–19 academic year. Sixteen (1%) California and nine (36%) North Carolina high schools responded and provided products, which were characterized by device type, e-liquid cartridge type, and brand.

Overall, 233 devices and 343 e-liquid cartridges were collected in California (Figure), and 176 devices and 267 e-liquid cartridges were collected in North Carolina. Pod mods were the most commonly collected devices in California (64%) and North Carolina (74%), and pod mod cartridges were the most commonly collected e-liquid cartridge type in California (80%) and North Carolina (81%). Among these devices and e-liquid cartridges, the three most commonly collected brands were Suorin (29%), SMOK (15%), and JUUL (14%) in California, and JUUL (48%), SMOK (16%), and Suorin (9%) in North Carolina.

**FIGURE:**
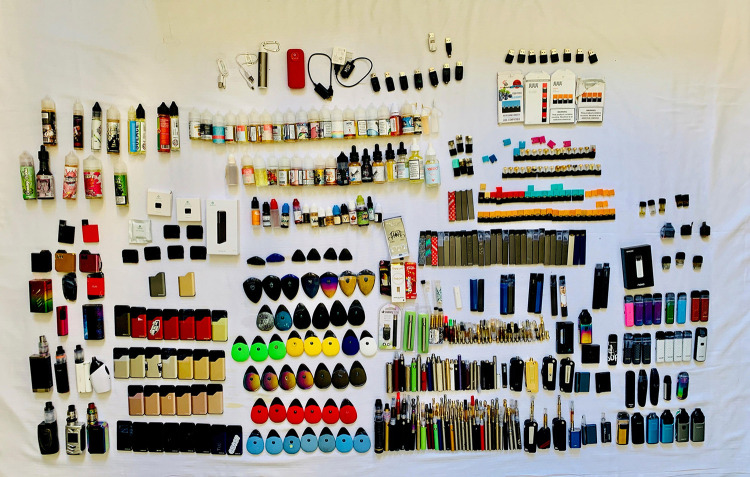
E-cigarette products confiscated from students by staff members or found on school grounds — 16 high schools, California, 2018–19 academic year

Approximately 1,000 e-cigarette, or vaping, products were collected from 25 high schools in California and North Carolina during the 2018–19 academic year. Pod mods, including JUUL, Suorin, and SMOK, were the three most commonly collected products, but variations in prevalence of collected device types and brands were observed between the two states. These differences could be attributed to brand popularity, affordability, or differing legal status of marijuana sales between states. For example, during the time of this study and currently, recreational and medicinal marijuana could be legally sold to persons aged ≥21 years in California and were thus present in society for potential indirect access by youths; in contrast, marijuana sales are currently illegal in North Carolina. Some types and brands of pod mod products are intended to be refilled by the user (e.g., Suorin and SMOK), which could include e-liquids containing nonnicotine substances such as marijuana; one third of current U.S. high school e-cigarette users report using marijuana in an e-cigarette (*4*).

The findings in this report are subject to at least three limitations. First, the response rates were low; thus, these findings might not be representative of all California and North Carolina schools. Second, not all schools retained confiscated e-cigarettes and other products, and some were discarded before the assessment. Moreover, school staff members had varying ability to accurately identify easily concealable products or those that resemble common objects like flash drives. Thus, the devices and products examined by investigators, as well as those confiscated and collected, might not be representative of all devices used by students. Finally, the contents of the confiscated products were not assessed.

School-based efforts to reduce and prevent tobacco product use are most effective when they are part of a comprehensive approach along with other evidence-based population-level strategies (*5*). School-level efforts could include adopting tobacco-free policies (including e-cigarettes) with enforcement measures that include access to resources and treatment for students, rather than punishment; implementing evidence-based curricula not sponsored by tobacco companies; and educating school staff members and parents about the changing product marketplace and known health risks of youth tobacco product use, including e-cigarettes (*5*).

## Footnotes

* https://www.cdc.gov/tobacco/basic_information/e-cigarettes/surgeon-general-advisory/index.html.

† https://www.cdc.gov/tobacco/basic_information/e-cigarettes/pdfs/ecigarette-or-vaping-products-visual-dictionary-508.pdf.

§ https://truthinitiative.org/sites/default/files/media/files/2019/03/Behind-the-explosive-growth-of-JUUL.pdf.
